# *In vitro* mechanistic studies and potential health benefits of a standardized bilberry extract in low mood and cognitive enhancement

**DOI:** 10.3389/fnut.2025.1630147

**Published:** 2025-07-31

**Authors:** Mehtap Kara, Gozde Hasbal-Celikok, Jacob Wilson, Pilar Gómez-Serranillos, Tugba Yilmaz-Ozden, Ezgi Öztas, Gul Özhan, Özge Sultan Zengin, Marta Sánchez Gómez-Serranillos, Claudia Owsianik, Fazle Rabbani, Merve Tunç, Nazlı Arda, Nazia M. Memon, Ikram Ujjan, Kenny Hawkins, Justine Davis, Gabriel Wilson, Giovanna Petrangolini, Amjad Khan

**Affiliations:** ^1^Department of Pharmaceutical Toxicology, Faculty of Pharmacy, Istanbul University, Istanbul, Türkiye; ^2^Department of Biochemistry, Faculty of Pharmacy, Istanbul University, Istanbul, Türkiye; ^3^Applied Science & Performance Institute, Tampa, FL, United States; ^4^Department of Pharmacology, Pharmacognosy and Botany, Faculty of Pharmacy, Complutense, University of Madrid, Madrid, Spain; ^5^Department of Psychiatry, Lady Reading Hospital, Peshawar, Pakistan; ^6^Department of Molecular Biology and Genetics, Institute of Graduate Studies in Sciences, Istanbul University, Istanbul, Türkiye; ^7^Department of Molecular Biology and Genetics, Faculty of Science, Istanbul University, Istanbul, Türkiye; ^8^Department of Pathology, Liaquat University of Medical and Health Sciences, Jamshoro, Pakistan; ^9^Medical Department, Indena SpA, Milan, Italy; ^10^Department of Biochemistry, Liaquat University of Medical and Health Sciences, Jamshoro, Pakistan; ^11^Nuffield Division of Clinical Laboratory Sciences (NDCLS), Radcliffe Department of Medicine, University of Oxford, Oxford, United Kingdom

**Keywords:** Bilberry extract, MirtoselectTM, mood, cognition, GABA-T, AChE, MAO-A

## Abstract

**Background:**

Low mood and cognitive impairments are multifactorial conditions often linked to oxidative stress, neurotransmitter imbalances, and neuroinflammation. Bilberry (*Vaccinium myrtillus L.*) extract, particularly rich in anthocyanins, has shown promising neuropharmacological properties in recent studies.

**Aims of the study:**

This study aimed to comprehensively evaluate the biochemical, antioxidant, and neuroprotective properties of a standardized bilberry extract (Mirtoselect™), alongside assessing its potential health benefits on mood and cognitive enhancement in a clinical setting.

**Methods:**

*In vitro* assays were conducted to explore the neuromodulatory, antioxidant, and cytoprotective properties of Bilberry extract. Enzyme inhibition assays targeted γ-Aminobutyric acid transaminase (GABA-T), monoamine oxidase A (MAO-A), and acetylcholinesterase (AChE), while GABA_*A*_ receptor binding was also evaluated. Antioxidant capacity was assessed using DPPH, ABTS, FRAP, ORAC, HORAC, and TAS assays. Neuroprotection was investigated using SH-SY5Y cells exposed to H_2_O_2_, assessing cell viability (MTT), membrane integrity (LDH release), and BDNF expression. Cytotoxicity was determined through the MTT assay in SH-SY5Y cells. A randomized, double-blind, placebo-controlled pilot clinical study was conducted on healthy adult subjects (*n* = 33) (aged 25–55 years) to evaluate the effects of Bilberry extract on mood (POMS) and cognitive function.

**Results:**

Bilberry extract demonstrated significant inhibition of GABA-T, MAO-A, and AChE, alongside moderate GABA_*A*_ receptor binding. It exhibited robust antioxidant activity in DPPH (EC_50_: 9.24 ± 0.22 μg/mL), ABTS (EC_50_: 12.70 ± 0.11 μg/mL), FRAP, ORAC, HORAC, and TAS assays. Neuroprotective effects included enhanced cell viability, reduced LDH release, and upregulation of BDNF in SH-SY5Y cells under oxidative stress. Cytotoxicity tests confirmed a favorable safety profile. In the pilot study, Bilberry extract supplementation significantly improved mood parameters, including reduced tension, depression, and confusion scores (*p* < 0.05) compared to placebo, with minimal adverse effects.

**Conclusion:**

Bilberry extract exhibits potent antioxidant, neuromodulatory, and neuroprotective properties, supporting its potential as a natural intervention for managing low mood and cognitive health. The favorable safety profile and preliminary clinical benefits warrant further research.

## 1 Introduction

Low mood and cognitive impairments are prevalent and complex conditions that can significantly impact an individual’s quality of life. These conditions are often linked to multiple interrelated factors, including oxidative stress, neurotransmitter imbalances, neuroinflammation, and impaired neuroplasticity. Conventional pharmacological treatments, such as antidepressants and cognitive enhancers, are widely used; however, they frequently come with limitations, including adverse effects, limited efficacy, and the risk of dependence (1). This has fueled growing interest in exploring safer, natural alternatives with neuroprotective and mood-enhancing properties (2–4).

**FIGURE 1:**
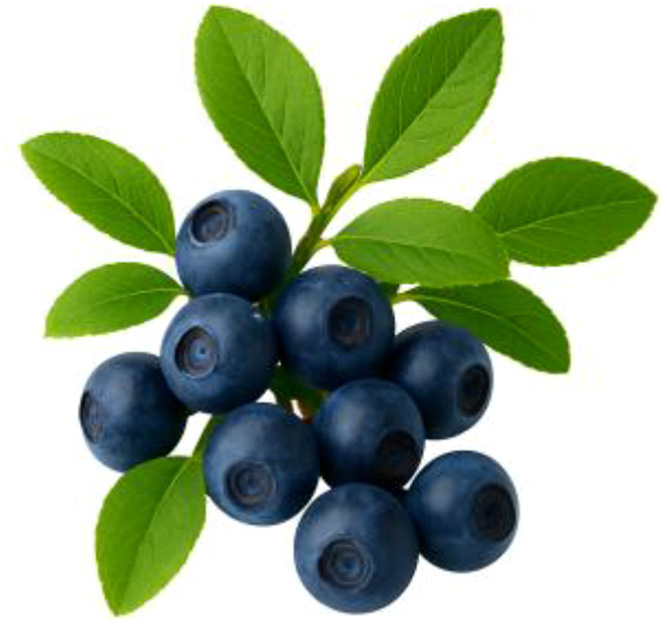
Bilberry fruit plant. Bilberry fruit contains high contents of bioactive compounds, including anthocyanins, flavonoids, and polyphenols.

A growing body of modern pharmacological evidence, coupled with a favorable safety profile, has amplified interest in natural compounds for mental health management (2–4). Plant-derived extract supplements, in particular, have garnered attention for their potential to support low mood and cognitive function (2–4). Among these botanicals, *Vaccinium myrtillus* L., commonly referred to as wild bilberry (Figure 1), has demonstrated promising effects in recent studies (5–9). Bilberry is a perennial plant that produces small, nutrient-rich berries widely recognized for their high content of bioactive compounds, including anthocyanins, flavonoids, and polyphenols (10). Anthocyanins, which constitute the majority of the phenolic content in bilberries, are recognized for their potent antioxidant, anti-inflammatory, and neuroprotective properties (10–13). Traditionally used in herbal medicine, bilberry has a long history of therapeutic application, dating back to the Middle Ages, where it was valued for managing inflammation, eye health, metabolic disorders, and skin conditions (10–13). Recent pharmacological research has further highlighted the neuropharmacological potential of bilberry, particularly in addressing mood-related disturbances and cognitive decline.

The potential benefits of bilberry for mood and cognition are thought to be mediated through its diverse bioactive profile, which may influence key molecular pathways associated with mental well-being. These mechanisms include modulation of gamma-aminobutyric acid (GABA) metabolism, inhibition of monoamine oxidase (MAO) activity, regulation of acetylcholine levels, and enhancement of antioxidant defenses. Such effects are directly linked to the polyphenolic content of bilberry, which can mitigate oxidative stress, support neurotransmitter balance, and promote neuronal survival.

Despite the traditional use and emerging scientific interest in bilberry, the precise mechanisms underlying its neuroprotective and mood-enhancing properties remain insufficiently characterized. This study aimed to bridge this knowledge gap by conducting a comprehensive *in vitro* mechanistic assessment of a standardized bilberry extract (Mirtoselect™), coupled with a pilot clinical evaluation. The extract demonstrated preliminary benefits on mood and cognitive function, along with an excellent safety profile, consistent with findings from numerous clinical trials (5–9). These results support its potential as a low-risk, natural option for managing low mood and cognitive health without the adverse effects commonly associated with conventional treatments.

## 2 Materials and methods

### 2.1 Bilberry extract

The bilberry extract used in this study was a highly standardized, food-grade extract derived exclusively from the wild bilberry (*Vaccinium myrtillus L.* Mirtoselect™, Indena S.p.A., Milan, Italy) (5–9, 14, 15), harvested from the forests of Northern and Eastern Europe. The extract was produced from frozen berries to preserve their bioactive components. Briefly, frozen bilberries were extracted with water/ethanol for 2 h at room temperature, then concentrated and finally purified through resin purification (water/ethanol elution), dried and milled to an extraction ratio of 80–130/1. The bilberry extract was standardized to contain ≥ 36% anthocyanins, expressed as Cyanidin 3-O-glucoside equivalents, as quantified by high-performance liquid chromatography (HPLC), ensuring consistent potency and a unique phytochemical profile (12, 16). This method also provides a specific fingerprint for quality control (5–9, 14, 15). Additionally, DNA barcoding was employed during quality control to guarantee authenticity, traceability, and consistency of the extract. The extract was provided in a fine, dark red-violet powder form, optimized for use in various formulations. For *in vitro* assays, the extract was dissolved in distilled water (dH_2_O) to achieve a stock solution of 1 mg/mL, with subsequent serial dilutions prepared to obtain the necessary concentrations for each assay.

For the pilot clinical study, the bilberry extract was formulated into vegetarian hard capsules, each containing 160 mg of the extract (supplement group). Placebo capsules, identical in appearance and composition except for the absence of the bilberry extract, contained rice flour with excipients (hypromellose, magnesium stearate, and chlorophyll). Both active and placebo capsules were manufactured (Nature’s Value Inc., Coram, NY) under identical conditions to maintain blinding integrity, ensuring that participants and investigators remained unaware of group assignments.

### 2.2 *In vitro* biochemical and enzymatic assays

All *in vitro* assays were performed in triplicate, using three freshly prepared samples for each experiment. The methods followed either previously published protocols or utilized commercially available assay kits. A positive and a negative control were included in every assay. Unless specified otherwise, all reagents were from commercial sources, and solutions were prepared in an aqueous buffer.

All *in vitro* procedures were conducted in accordance with institutional biosafety guidelines. Biohazardous materials, including cell culture waste and chemical reagents, were disposed of using certified biohazard waste containers and autoclaving or chemical neutralization procedures, as appropriate.

#### 2.2.1 Human SHSY5Y cell culture

The human SH-SY5Y neuroblastoma cell line (American Type Culture Collection (ATCC, # CRL-2266™) (17, 18) was maintained in DMEM/F-12 medium supplemented with 10% fetal bovine serum (FBS) and 100 U/mL of penicillin-streptomycin, incubated at 37°C in a humidified environment with 5% CO2. Cells were passaged every 3–4 days once they reached confluence. Seeding densities were adjusted according to the specific requirements of each experiment. The extract was administered to the cell cultures for a 24-h exposure period.

#### 2.2.2 Neurotransmitter-modulating assays

##### 2.2.2.1 γ-Aminobutyrate transaminase (GABA-T) inhibition assay

The GABA-T inhibition assay was carried out as previously reported (16, 17), using a commercially available kit following the manufacturer’s instructions (BRSC, University at Buffalo, USA, #E-134) (16). The control used were dH_2_O as negative and GABA-T substrate as positive. The Optical density (OD) was measured at 492 nm (OD492) using a plate reader. GABA-T enzyme activity was determined by subtracting the OD of the negative control wells (ODC) from the OD of the positive reaction wells (ODR). A linear regression analysis was conducted to evaluate the concentration-dependent inhibitory effect of bilberry extract on GABA-T activity, and IC_50_ values were derived from the dose-response curves.

##### 2.2.2.2 γ-Aminobutyric acid type A (GABA_*A*_) receptor binding assay

The GABA_*A*_ receptor binding assay was adapted from the method by Heaulme et al. as previously described (16, 18), using rat brain tissue as the receptor source. [3H]-Muscimol (6 nM) served as the radioligand, and SR95531 (31.6 μM) was used to assess non-specific binding. Bilberry extract was solubilized in Milli-Q^®^ H_2_O and diluted further. Membranes were thawed, centrifuged, and resuspended in assay buffer for a 30-min incubation at 37°C. The assay was terminated by filtering the samples onto GF/C plates, followed by washing with ice-cold buffer. Bound radioactivity was measured using a microplate reader. Specific Mirtoselect™ binding was calculated as the difference between total and non-specific binding. IC_50_ values were determined through non-linear regression analysis of competition curves using GraphPad Prism.

##### 2.2.2.3 Monoamine oxidase A (MAO-A) inhibition assay

The MAO-A inhibition assay was performed using the MAO-A Inhibitor Screening Kit (OxiSelect™, CellBiolabs, San Diego, USA, #STA-324), which utilizes recombinant human MAO-A enzyme expressed in *Escherichia coli*, with a reported purity of ≥ 90% as determined by SDS-PAGE, according to the manufacturer’s specifications. The assay followed the manufacturer’s instructions and previously published protocols (16, 17). In summary, 50 μL of the kit standard, control, and bilberry extract solutions at various concentrations were dispensed into separate wells of a microtiter plate. Next, 50 μL of the assay working solution was added to each well, and the contents were mixed thoroughly. The plate was then incubated for 60 min at room temperature, protected from light. Following the incubation, absorbance was recorded using a microplate reader (Biotek, Epoch™, Vermont, USA) within the 540–570 nm wavelength range. Results were expressed as a percentage of relative inhibition.

##### 2.2.2.4 Acetylcholinesterase (AChE) inhibition assay

The AChE inhibition assay was performed following a previously established method (16). In brief, 20 μL of bilberry extract at different concentrations was mixed with 180 μL of Ellman’s reagent (DTNB - Sigma #D8130, and 955 mM ATChI - Sigma #A5751) in 0.1 M phosphate buffer at pH 7.5. To initiate the reaction, 50 μL of AChE solution (Sigma #C3389; 0.5 U/mL) was added. This enzyme is derived from *Electrophorus electricus* (electric eel) and has a reported purity of ≥ 50% based on enzymatic activity, according to the manufacturer. The reaction progress was tracked by monitoring the absorbance change at 412 nm over a 10-min period. Control experiments were performed by replacing the extract with 20 μL of the solvent. Galantamine (Sigma #G1660) was used as the reference standard inhibitor for AChE. The percentage of AChE inhibition was calculated using the formula, previously described (16). IC_50_ values were calculated by linear regression analysis from dose-response curves.

#### 2.2.3 Protection of SH-SY5Y cells against H_2_O_2_-induced oxidative stress assays

##### 2.2.3.1 MTT [3-(4,5-Dimethylthiazol-2-yl)-2,5-Diphenyl Tetrazolium Bromide] detection

The MTT assay was conducted following a previously established protocol (16, 17). In summary, SH-SY5Y cells (1 × 10^4 cells/well) were co-treated with 150 μM H_2_O_2_ and various concentrations of bilberry extract for an additional 24 h (16, 17). MTT solution (5 mg/mL) was then incubated with cells at 37°C for 3 h. The conversion of MTT to formazan, which reflects metabolic activity, was quantified by measuring the absorbance at 590 nm using a Biotek Epoch™ microplate spectrophotometer (Agilent, Santa Clara, CA, USA). The same assay without the cotreatment with H_2_O_2_, also gave data on the cytotoxic effect of bilberry extract on SH-SY5Y cells, indicated by reduced cell viability and metabolic activity.

##### 2.2.3.2 Lactate dehydrogenase (LDH) release

The LDH release assay was performed based on a previously reported method (16, 17). SH-SY5Y cells (1 × 10^4 cells/well) were co-treated with 150 μM H_2_O_2_ and varying concentrations of bilberry extract for 24 h. LDH release, which indicates membrane damage, was measured using the LDH cytotoxicity assay kit (Roche, #11644793001). Triton X-100 (1%) was used as a positive control. Optical density was assessed at 495 nm using a microplate reader, and the percentage of LDH release was calculated based on a linear regression curve.

#### 2.2.4 Cell-free antioxidant activity assays

##### 2.2.4.1 DPPH (2,2-Diphenyl-1-picrylhydrazyl) radical scavenging activity assay

The DPPH radical scavenging assay was conducted using a previously reported method (16, 17, 19). In brief, 10 μL of bilberry extract at various concentrations was combined with 240 μL of 0.1 mM DPPH radical solution. The incubated mixture after 30 min expressed a decrease in absorbance, indicating the reduction of DPPH radicals measured at 517 nm. Quercetin was utilized as a standard antioxidant, while a control was prepared using the methanol instead of the extract. The percentage of DPPH radical scavenging activity was calculated as previously reported (16). Half-maximal effective concentration (EC_50_) values for bilberry extract were determined from the percentage inhibition of DPPH radicals.

##### 2.2.4.2 ABTS [2,2′-Azinobis-(3-ethylbenzothiazoline-6-sulfonic acid)] radical scavenging activity assay

The ABTS radical scavenging activity of bilberry extract was evaluated using a modified method from Re et al. as previously described (16, 17, 20). A 5 μL sample of bilberry extract at various concentrations was mixed with 245 μL of an ABTS working solution (concentration 2.45 mM). After a 6-min incubation, decolorization was measured at 734 nm using a spectrophotometer (Biotek, Epoch™, Vermont, USA). Quercetin served as the standard, and a control was prepared using the solvent (ethanol). The ABTS radical scavenging activity was calculated as previously reported (16). IC_50_ values were derived from the percentage of ABTS radical inhibition.

##### 2.2.4.3 Ferric reducing antioxidant power (FRAP) assay

The antioxidant activity of bilberry extract was evaluated using the ferric reducing antioxidant power (FRAP) assay, following a modified method described by Avan et al. (19, 21). Samples (110 μL) were combined with 900 μL of the FRAP reagent and incubated at 37°C for 10 min. Absorbance was measured at 595 nm using a SPECTROstar™ Nano Microplate Reader (BMG Labtech, Ortenberg, Germany). A calibration curve was generated using five concentrations of FeSO_4_⋅7H_2_O (1, 0.8, 0.4, 0.1, 0.05 μmol), and results were expressed as μmol equivalents of Fe(II) per gram of sample solution.

##### 2.2.4.4 Oxygen radical antioxidant capacity (ORAC) assay

The ORAC assay was conducted as previously described (16, 17), using a commercial kit (BQC, Asturias, Spain, #KF01004). Briefly, 15 μL of each standard and bilberry extract solution was incubated with 90 μL of reagent B at 37°C for 15 min. Then, 45 μL of reagent C was added to each well. Fluorescence was measured to evaluate antioxidant activity, reflecting the sample’s ability to inhibit the reduction in fluorescence caused by oxidative degradation of a fluorescent probe, using a fluorescent plate reader set at excitation/emission wavelengths of 485 nm/528–538 nm (FLx800, BioTek, Agilent, Santa Clara, CA, USA).

##### 2.2.4.5 Hydroxyl radical antioxidant capacity (HORAC) assay

The assay was carried out using a commercial kit (MyBioSource, Inc., USA, #MBS48047) as previously described (16, 17). Bilberry extract was diluted with a methanol/water mixture (70:30) and combined with fluorescein. This mixture was added to all wells, including those containing samples and standards. After a 30-min incubation at 37°C, hydroxyl radicals and Fenton reagent were added. The plate was read every minute for 45 min at an excitation wavelength of 488 nm and an emission wavelength of 515 nm. Results were compared to a standard curve of gallic acid to express results in gallic acid equivalents (GAE) per milligram of sample.

##### 2.2.4.6 Total antioxidant status (TAS) assay

The TAS assay was conducted as described previously (16), using a commercial kit (Elabscience™, USA, #E-BC-K801-M). Bilberry extract samples were diluted in 60% ethanol and mixed with a buffer solution in the wells. An initial absorbance measurement (A1) was taken. A chromogenic solution containing oxidized ABTS was then added, and the mixture was incubated for 5 min at 37°C. A second absorbance measurement (A2) was recorded at 660 nm. The results were analyzed against a Trolox standard, providing total antioxidant capacity in mmol of Trolox equivalents per kilogram of wet weight.

#### 2.2.5 Total phenolic content assay

The total phenolic content (TPC) in bilberry extract was determined using the Folin–Ciocalteau method (16). First, a reaction mixture was prepared by combining 10 μL of bilberry extract (1 mg/mL), 200 μL of Folin–Ciocalteu reagent, and incubated for 5 min to allow initial reaction between phenolics and the reagent (22, 23). Following this, 90 μL of 7% sodium carbonate solution was added. Subsequently, the mixture was incubated at 37°C in the dark, and after 40 min, the absorbance was recorded at 595 nm using a SPECTROstar™ Nano Microplate Reader (BMG Labtech, Ortenberg, Germany). A calibration curve was constructed using gallic acid as the standard. The results are expressed as micrograms of gallic acid equivalents (GAE) per mg of extract, with values averaged from triplicate assays. Trolox was used as a positive control to confirm assay performance.

### 2.3 Clinical pilot study

The randomized, double-blind, placebo-controlled pilot clinical study was conducted to evaluate the potential health benefits of standardized Bilberry extract (Mirtoselect™) on cognitive enhancement, as assessed through standardized cognitive performance tests conducted during the acute phase at 60 and 180 min post-intake and after 4 weeks of supplementation. Secondary endpoints included the evaluation of potential benefits on mood states and assessment of the safety profile of the supplementation.

The study was carried out at the Applied Science and Performance Institute (ASPI Labs), Florida, USA. The protocol received approval from an external Institutional Review Board (Advarra IRB, ID: Pro00074459, September 2023) and was conducted in accordance with the Declaration of Helsinki, International Conference on Harmonization Good Clinical Practice guidelines (ICH E6-R2), and applicable regulatory requirements. The study was registered in ClinicalTrials.gov trials registry (ID: NCT06309914).

Eligible participants included healthy adults aged 25–55 years, capable of providing informed consent and complying with study procedures. Exclusion criteria encompassed individuals with cognitive impairment, significant medical conditions (cardiovascular, neurological, gastrointestinal, metabolic disorders), history of malignancy, pregnancy, substance abuse, and use of medications affecting digestion.

Following online pre-screening, eligible participants across the United States were randomized (1:1) to receive either the Bilberry extract supplement or a placebo. Randomization was computer-generated, with blinding maintained for participants, investigators, and outcome assessors.

Participants attended baseline visits in a fasted state, during which cognitive performance and mood state were assessed. Acute effects were measured at 60 and 180 min post-supplementation with 2 capsules (320 mg total) of Mirtoselect™ or placebo. Participants were then instructed to consume 2 capsules daily for 4 weeks. After this period, outcome measures were reassessed in the fasted state.

Cognitive performance was evaluated using the CNS Vital Signs computerized testing system (Morrisville, NC, USA), assessing visual and verbal memory, motor speed, processing speed, cognitive flexibility, executive function, and vigilance. Mood state was measured using the Abbreviated Profile of Mood States (POMS), a validated 40-item scale providing subscale scores and a total mood disturbance (TMD) score.

Adverse events were monitored and recorded throughout the study, with severity and relatedness to the study product determined by the investigators. Compliance was assessed through capsule count and self-reported adherence.

Primary and secondary outcomes were analyzed using generalized linear mixed models, with time (baseline, acute, and 4 weeks) as a repeated factor and treatment group as a between-subject factor. Pairwise comparisons were conducted with Bonferroni correction for multiple comparisons. Statistical significance was defined as *p* < 0.05.

### 2.4 Statistical analysis

*In vitro* activity assays data are presented as mean ± standard deviation (SD). Statistical analyses were performed using one-way analysis of variance (ANOVA) followed by *post hoc* Dunnett’s multiple comparison test, using SPSS version 20 (IBM SPSS Inc., New York, NY, USA). A *p*-value of < 0.05 was considered statistically significant.

For the clinical pilot study, the statistical analysis was designed to assess the significance of primary and secondary outcomes using generalized linear mixed models (GLMM). In these models, time (baseline, 60 min, 180 min, and Week 4) was treated as a categorical repeated measures variable (within-subject factor), while treatment groups (Placebo and Bilberry extract) were treated as a between-subjects factor. Subjects were included as a random factor to account for individual variability.

Pairwise comparisons of marginal means were conducted using sequential Bonferroni correction to adjust for multiple comparisons, ensuring rigorous statistical control. The model accounted for interactions between time and treatment group, with statistical significance established at *p* < 0.05 for all analyses.

All statistical analyses were performed using SPSS version 20 (IBM SPSS Inc., New York, NY, USA).

## 3 Results

### 3.1 *In vitro* mechanistic evaluation

#### 3.1.1 GABA-T inhibition assay

**FIGURE 2:**
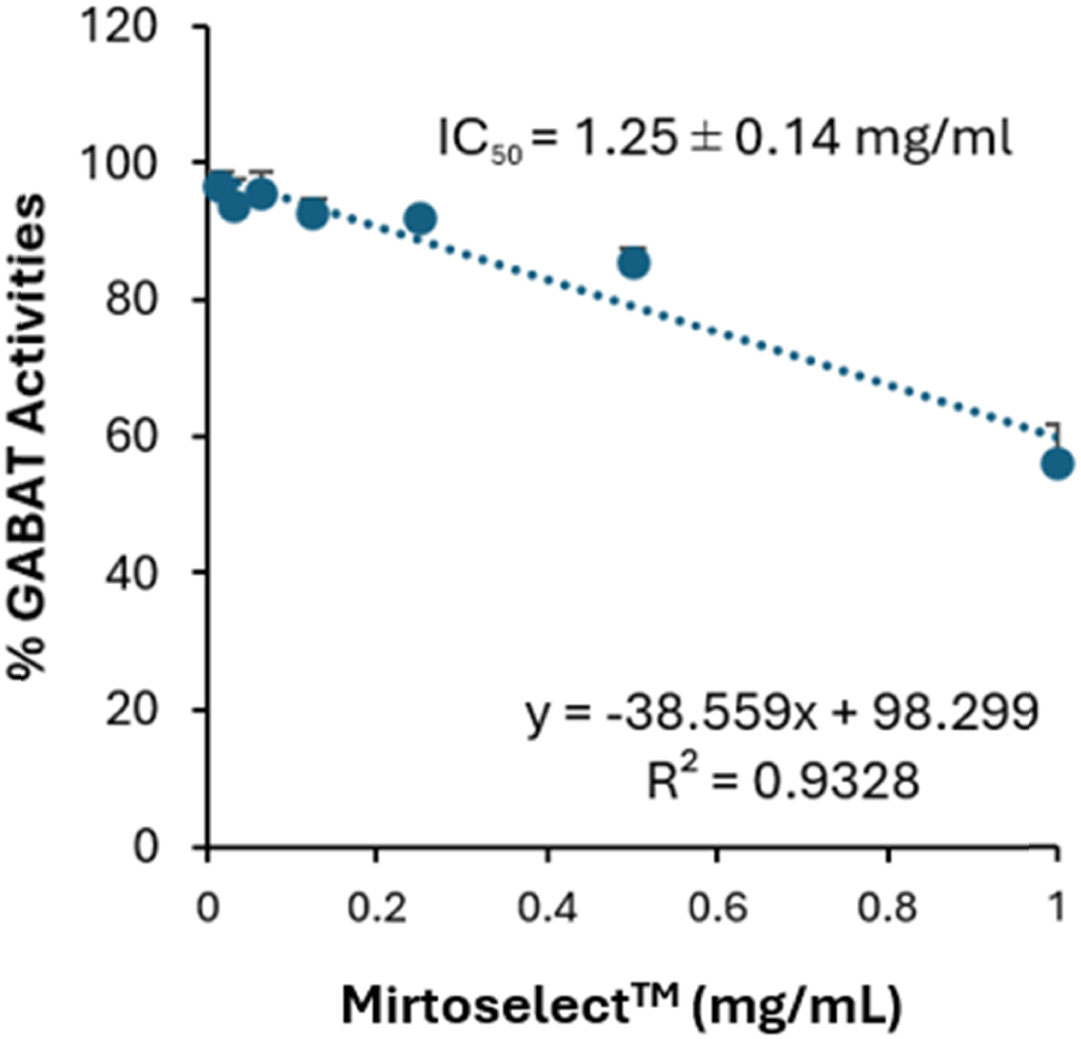
Inhibitory effect of bilberry extract (Mirtoselect™) on GABA-T enzyme activity. Error bars represent standard deviations from the mean of three assays.

In the GABA-T enzyme inhibition assay, bilberry extract exhibited a concentration-dependent reduction in enzyme activity (Figure 2). The IC_50_ value was determined to be 1.25 ± 0.14 mg/mL, indicating effective inhibition of GABA-T at this concentration. The linear regression analysis (R^2^ = 0.9328) demonstrates a strong correlation between extract’s concentration and GABA-T activity. This mechanism aligns with the bilberry extract’s potential to influence pathways associated with emotional regulation.

#### 3.1.2 GABA_*A*_ receptor binding assay

**FIGURE 3:**
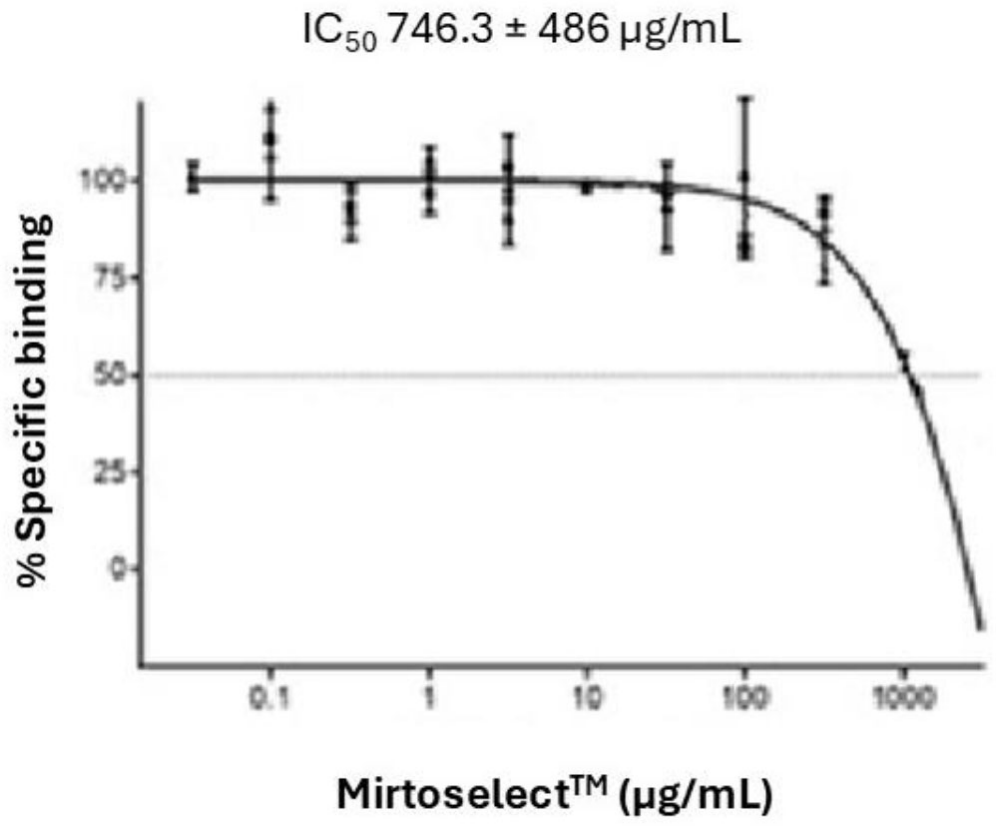
Binding activity of bilberry extract (Mirtoselect™) to GABA_*A*_ receptors. Error bars represent standard deviations from the mean of three assays.

The GABA_*A*_ receptor binding assay of the bilberry extract revealed a concentration-dependent reduction in specific binding, with an IC_50_ value of 746.3 ± 486 μg/mL (Figure 3). These findings suggest that bilberry extract exhibits moderate affinity for GABA_*A*_ receptor sites.

#### 3.1.3 MAO-A inhibition assay

**FIGURE 4:**
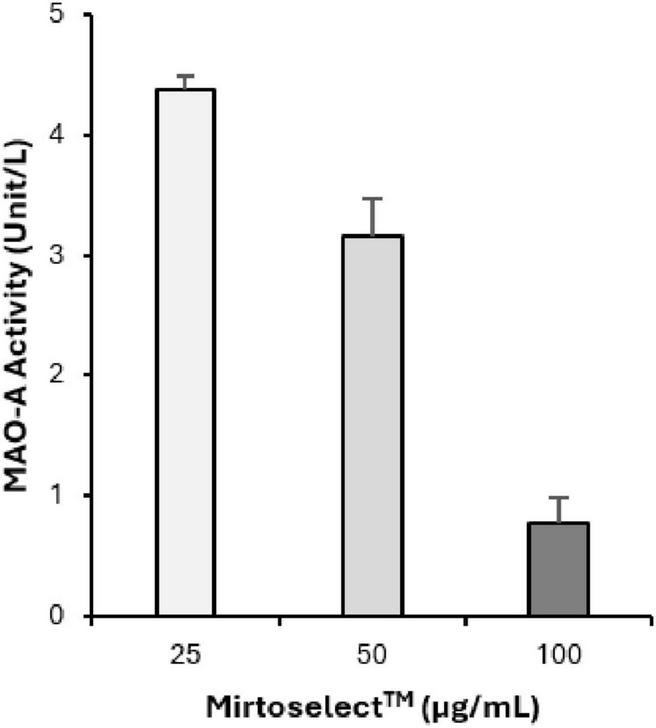
Inhibitory effect of bilberry extract (Mirtoselect™) on MAO-A enzyme activity. Error bars represent standard deviations from the mean of three assays.

The MAO-A enzyme inhibition assay demonstrates that bilberry extract exhibits a dose-dependent inhibitory effect on MAO-A activity (Figure 4). The concentrations tested ranged from 25 to 100 μg/mL. These results suggest that bilberry extract possesses MAO-A inhibitory effects, highlighting its potential role in modulating monoamine levels, which could contribute to mood regulation.

#### 3.1.4 AChE inhibition assay

**FIGURE 5:**
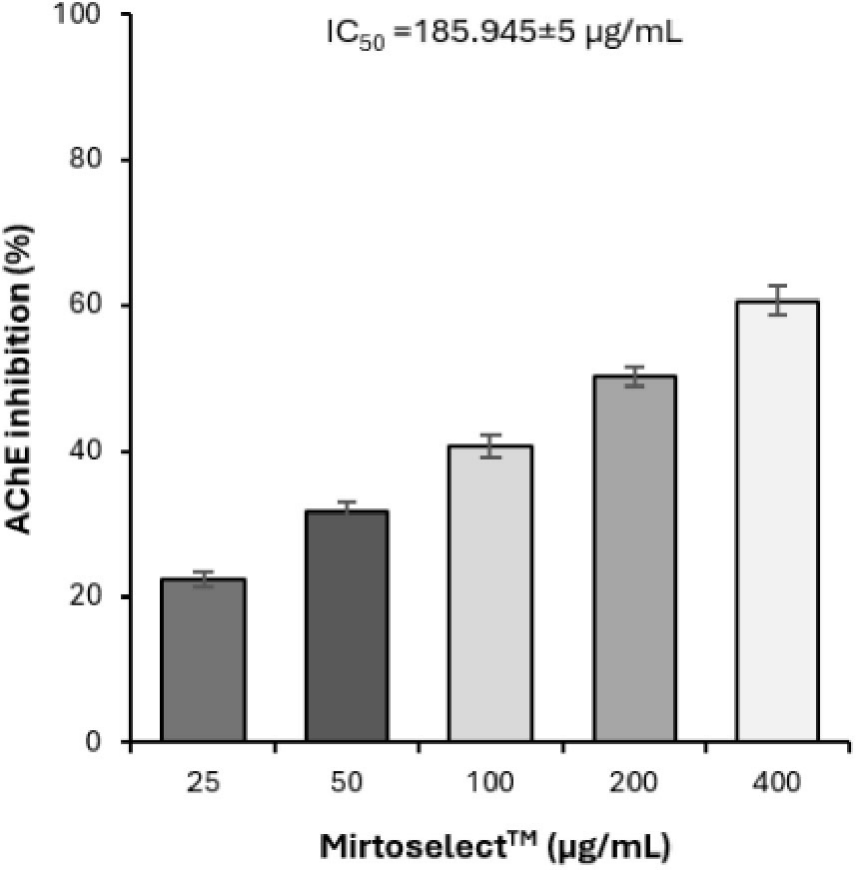
Inhibitory effect of bilberry extract (Mirtoselect™) on AChE enzyme activity. Error bars represent standard deviations from the mean of three assays.

The AChE enzyme inhibition assay demonstrated that bilberry extract inhibits AChE activity in a dose-dependent manner, with an IC_50_ value of 185.94 ± 5 μg/mL (Figure 5), indicating moderate activity. These results suggest that bilberry extract may help increase acetylcholine levels by preventing its breakdown, which could play a role in managing cholinergic signaling and potentially improving cognitive functions. Galantamine, which was used as a standard inhibitor, demonstrated an IC_50_ value of 0.806 ± 0.014 μg/mL.

#### 3.1.5 SH-SY5Y cells neuroprotective activity assay

**FIGURE 6:**
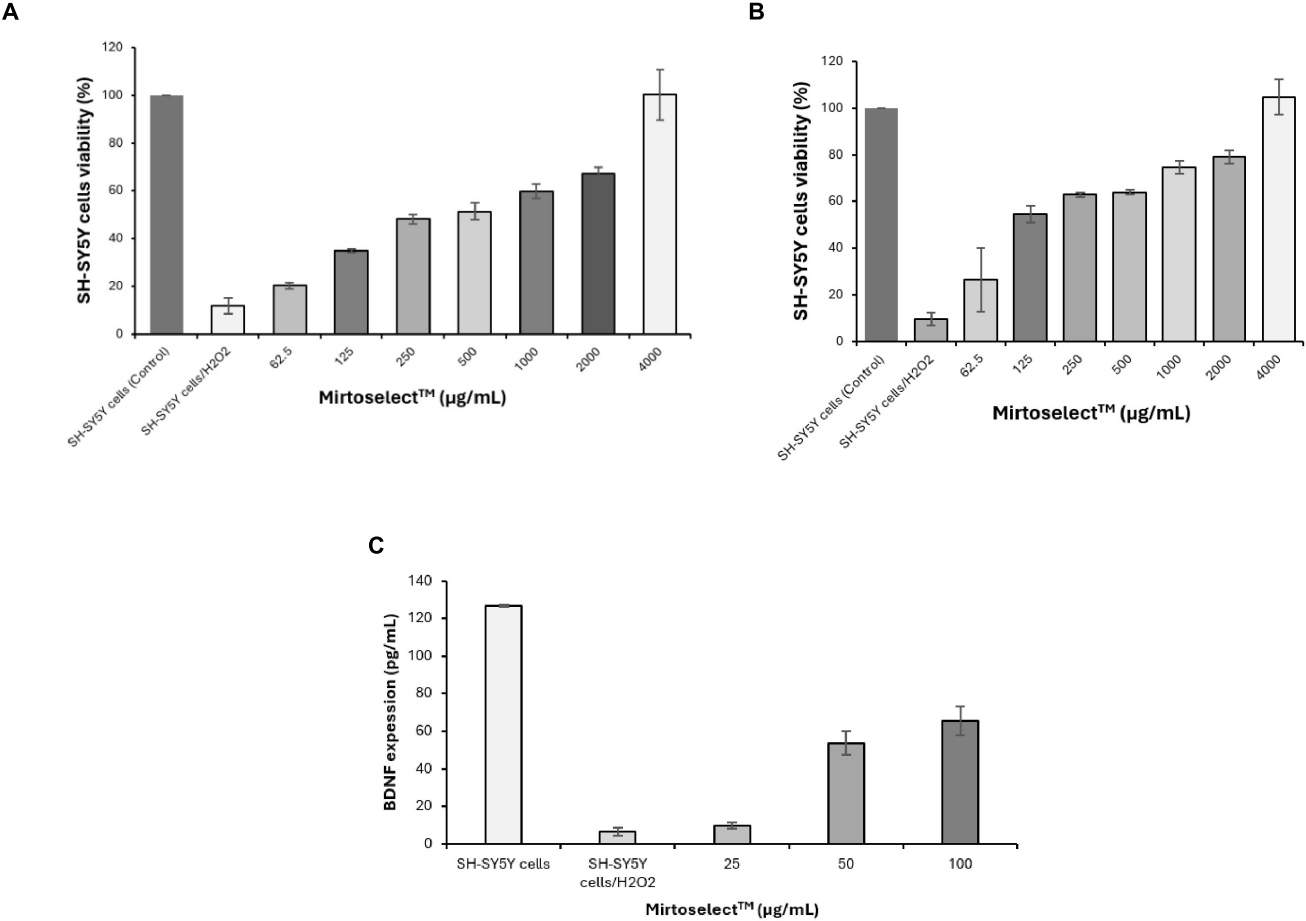
Neuroprotective effect of bilberry extract (Mirtoselect™) on SH-SY5Y neuronal cells exposed to H_2_O_2_-induced oxidative stress. **(a)** MTT assay, **(b)** LDH release assay, and **(c)** SH-SY5Y cells BDNF expression level assay. Error bars represent standard deviations from the mean of three assays.

Both the MTT (Figure 6a) and LDH release assays (Figure 6b) demonstrated that bilberry extract effectively protected SH-SY5Y neuronal cells from H_2_O_2_-induced oxidative stress in a concentration-dependent manner. These findings suggest that bilberry extract has significant neuronal cytoprotective potential, capable of mitigating oxidative damage associated with neurodegeneration. Additionally, in a separate assay, bilberry extract enhanced the expression of BDNF in SH-SY5Y cells exposed to H_2_O_2_-induced oxidative stress (Figure 6c), further reinforcing its neuroprotective properties. The upregulation of BDNF is particularly relevant, as it plays a critical role in neuronal survival, plasticity, and cognitive function. These results underscore the potential of bilberry extract as a promising candidate for improving mental well-being and addressing mood disorders associated with oxidative stress and BDNF dysregulation.

The potent antioxidant effects of bilberry extract were further assessed through a series of chemical tests, as detailed below.

**FIGURE 7:**
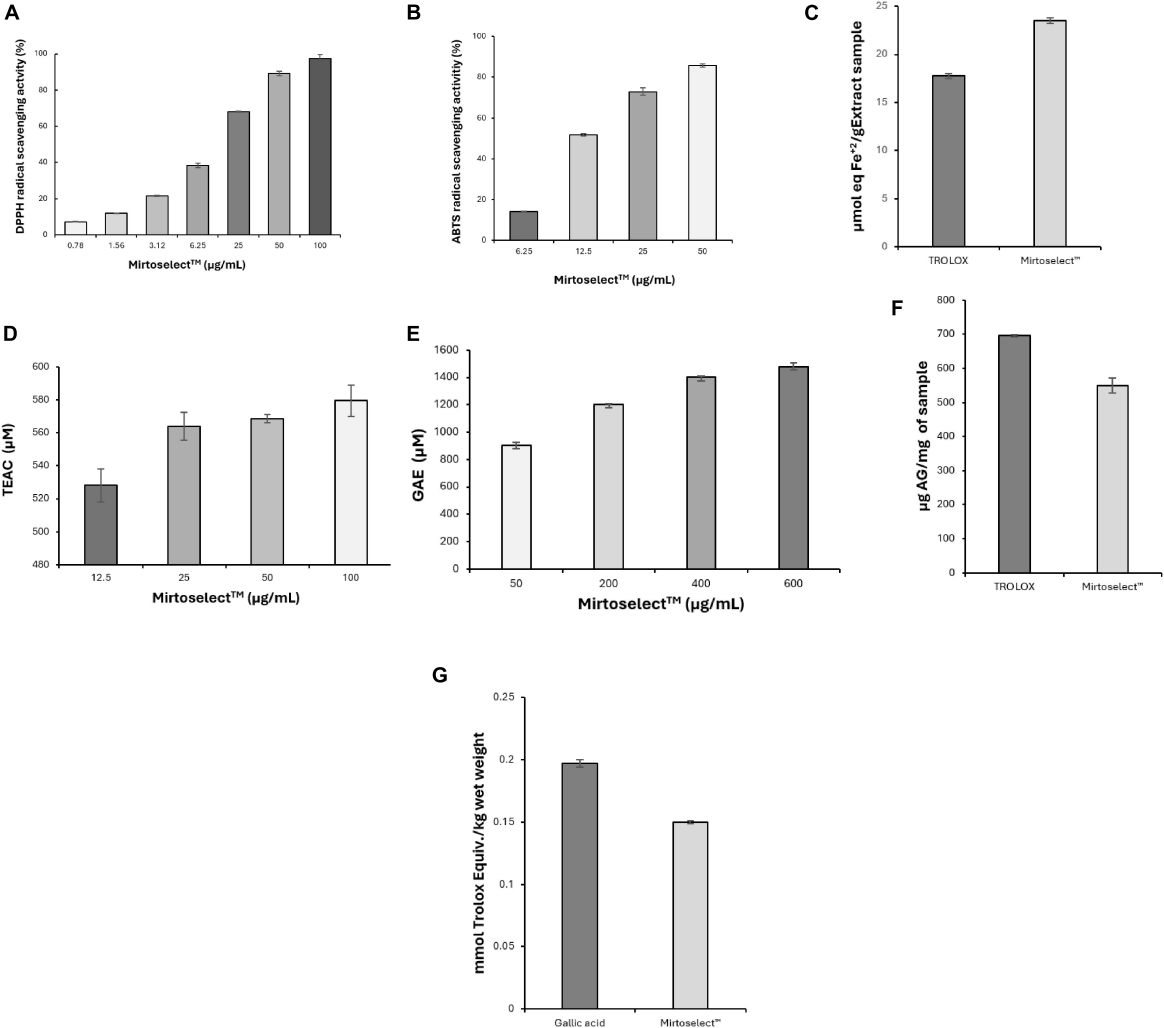
Evaluation of the antioxidant activity of bilberry extract (Mirtoselect™) in various chemical assays. **(a)** DPPH radical-scavenging assay, **(b)** ABTS radical-scavenging assay, **(c)** FRAP assay, **(d)** ORAC assay, **(e)** HORAC assay, **(f)** total phenolic contents assay and **(g)** total antioxidant status assay. Error bars represent standard deviations from the mean of three assays.

The DPPH radical-scavenging activity of bilberry extract revealed a strong and dose-dependent antioxidant effect (Figure 7a). Bilberry extract effectively neutralized DPPH radicals, demonstrating its high capacity to donate electrons or hydrogen atoms. The EC_50_ value was determined to be 9.24 ± 0.22 μg/mL, highlighting its potent free-radical neutralization capability. Quercetin, used as a standard antioxidant, exhibited an EC_50_ value of 3.86 ± 0.28 μg/mL.

Consistent with the DPPH results, the ABTS assay demonstrated that bilberry extract displayed significant antioxidant activity by efficiently quenching ABTS radicals (Figure 7b). The activity was dose-responsive, with an EC_50_ value of 12.70 ± 0.11 μg/mL, underscoring its potent radical-scavenging capability. Quercetin was used as a standard antioxidant in the ABTS radical scavenging assay and was determined to have an EC_50_ value of 3.92 ± 0.08 μg/mL.

In the FRAP assay, bilberry extract demonstrated significant ferric-reducing antioxidant power, showing its strong electron-donating capacity (Figure 7c). This activity reflects its ability to efficiently reduce ferric ions (Fe^3+^) to their ferrous state (Fe^2+^), a critical mechanism for combating oxidative stress.

The ORAC assay revealed that bilberry extract effectively neutralized peroxyl radicals, a primary source of oxidative stress in biological systems, even at low concentrations (Figure 7d), reinforcing its robust antioxidant potential.

In addition to neutralizing reactive oxygen species bilberry extract demonstrated significant hydroxyl radical scavenging activity in the HORAC assay, with its antioxidant capacity increasing in a concentration-dependent manner (Figure 7e).

The total phenolic content of bilberry extract was found to be high (Figure 7f), correlating strongly with its observed antioxidant activity across multiple assays. Phenolic compounds are known contributors to the antioxidant properties of plant-based extracts, providing additional evidence for the bioactive compounds responsible for bilberry extract’s efficacy.

Finally, the TAS assay revealed that the high TAS of bilberry extract (Figure 7g) provides a comprehensive measure of its overall antioxidant capacity, effectively capturing the synergistic effects of its various bioactive components.

Overall, the results from these assays demonstrate the strong and multifaceted antioxidant properties of bilberry extract, confirming its ability to neutralize a broad spectrum of reactive oxygen species and support redox balance. These findings suggest that bilberry extract may mitigate oxidative damage associated with low mood and enhance mood regulation.

#### 3.1.6 Cytotoxicity effect evaluation in SH-SY5Y cells

The results of the MTT cytotoxicity assay indicate that bilberry extract did not show cytotoxic effects on SH-SY5Y cells within a concentration range of 500–4000 μg/mL (data not shown). Cell viability remained high and similar to that of the control group (culture medium only), confirming the favorable safety profile of bilberry extract in neuronal cells.

### 3.2 Clinical pilot study

A total of 41 participants were enrolled in the study and were randomized to receive either the Bilberry extract supplement (Mirtoselect™) (*n* = 21) or placebo (*n* = 20). Of these, 17 participants in the Bilberry extract group and 16 in the placebo group completed the study. Participants who did not complete the study were lost to follow-up or withdrew due to personal reasons.

#### 3.2.1 Cognitive performance

**FIGURE 8:**
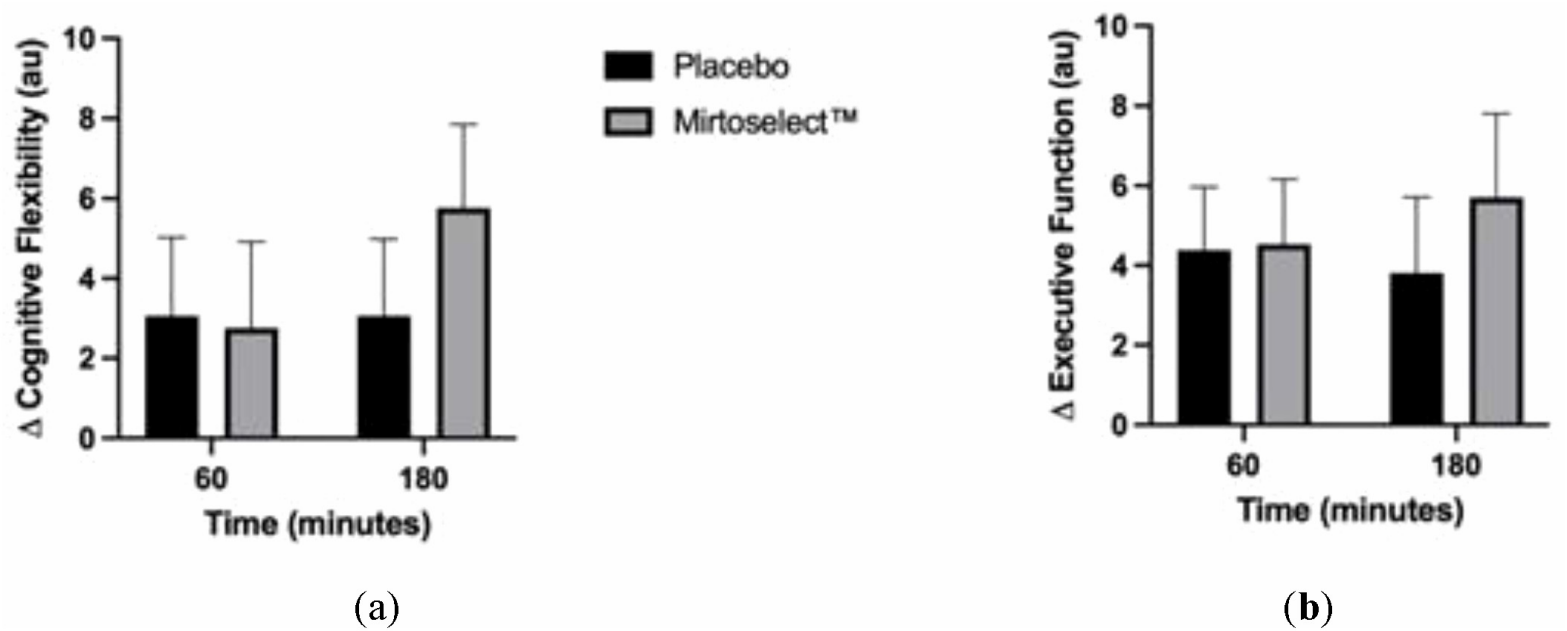
**(a)** Delta changes in cognitive flexibility after acute bilberry extract (Mirtoselect™) supplementation (at 60–180 min); **(b)** Delta changes in executive function after acute bilberry extract (Mirtoselect™) supplementation (at 60–180 min). Values are reported as means ± SEM.

The analysis of cognitive performance revealed no statistically significant interactions between treatment groups (Bilberry extract vs. placebo) across the acute (60 and 180 min) or 4-week supplementation periods. However, a tendency for improved cognitive flexibility was observed in the Bilberry extract group at 180 min post-supplementation, defined as enhanced ability to recognize set shifting (mental flexibility) and abstraction (rules, categories) (Figure 8a). Additionally, there was a trend toward improved executive function, characterized by the ability to sequence and manage multiple tasks, in the Bilberry extract group at 180 min (Figure 8b). These trends did not reach statistical significance.

#### 3.2.2 Mood states

**FIGURE 9:**
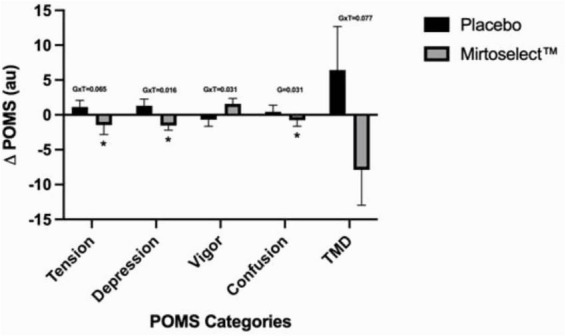
Bilberry extract (Mirtoselect™) 4-week (pre to post) Delta changes in POMS values. Values are reported as means ± SEM. Total mood disturbance (TMD). GxT, group by time interaction *p*-values; G, Main effect for group *p*-value; *p < 0.05 vs placebo, using pairwise comparisons.

Regarding mood states, no significant changes were observed immediately following a single acute dosage. However, after 4 weeks of supplementation, several significant group-by-time interactions were detected. These included reductions in depression (*p* = 0.016), improvements in vigor (*p* = 0.031), and a trend for reduced tension (*p* = 0.065) and total mood disturbance (TMD) score (*p* = 0.077). A main group effect was observed for confusion (*p* = 0.031). Pairwise comparisons further revealed that the Bilberry extract group experienced significantly reduced tension (*p* = 0.041), depression (*p* = 0.029), and confusion (*p* = 0.031) compared to the placebo group (Figure 9).

#### 3.2.3 Adverse events

Two adverse events were reported during the study, both occurring in the Bilberry extract group. One participant reported a mild headache, while another experienced a sensation of feeling clammy. Both events were self-limiting, resolved without intervention, and were considered unlikely to be related to the study product. No serious adverse events were reported throughout the study period.

## 4 Discussion

The findings of this study underscore the potential of bilberry extract as a multifunctional natural tool for addressing low mood, cognitive support, and neuroprotection. By leveraging its anthocyanin-rich composition, Bilberry extract demonstrated robust activity across multiple biochemical pathways critical to mood regulation and cognitive health, confirmed in a pilot human study.

The GABA-T inhibition assay revealed that Bilberry extract effectively reduces GABA-T activity in a dose-dependent manner, indicating significant inhibitory potency. Notably, this study is the first to report the GABA-T inhibitory activity of bilberry extract, highlighting its novel potential in neuromodulation. GABA-T is a key enzyme responsible for the breakdown of GABA, a principal inhibitory neurotransmitter in the central nervous system. Its inhibition by Bilberry extract could potentially elevate GABA levels in the synaptic cleft, thereby enhancing inhibitory neurotransmission and potentially reducing symptoms associated with anxiety and low mood. In addition to inhibiting GABA-T, Bilberry extract demonstrated binding affinity to GABA_*A*_ receptor sites, indicating interaction at higher concentrations. GABA_*A*_ receptors mediate the fast inhibitory effects of GABA by increasing chloride ion influx, which hyperpolarizes neurons and reduces excitability. While the binding effect is weak, it may still complement the increased GABA availability resulting from GABA-T inhibition, contributing to a broader neuromodulatory effect. Together, these dual actions suggest that Bilberry extract engages multiple pathways within the GABAergic system, offering potential benefits for managing low mood and emotional dysregulation.

The inhibition of MAO-A by Bilberry extract presents another significant finding. MAO-A plays a pivotal role in the degradation of neurotransmitters such as serotonin and dopamine, which are heavily implicated in mood regulation. The MAO-A inhibitory effects observed in this study suggest that Bilberry extract could elevate monoamine levels, potentially alleviating symptoms of low mood. A prior study demonstrated that blueberry juice inhibits MAO-A activity in a dose-dependent manner, further validating the potential of berry-based interventions in modulating monoamine metabolism (24). Additionally, it has been reported that berry fruit constituents, including anthocyanins, anthocyanidins, proanthocyanidins, and phenolic metabolites, inhibit both MAO-A and MAO-B (25), suggesting a broader mechanism that could further enhance neurotransmitter availability and mood regulation. The anthocyanin-rich profile of Bilberry extract likely underpins these activities, reinforcing its role as a natural agent for targeting biochemical pathways associated with emotional well-being. Together, these findings contribute to a growing body of evidence supporting the potential of berry-derived products in mental health management. Additionally, the AChE inhibition assay results, consistent with findings from previous studies (26, 27), underscore Bilberry extract’s potential in supporting cognitive functions. By inhibiting acetylcholinesterase, Bilberry extract may help sustain acetylcholine levels, which is particularly critical in conditions like age-related cognitive decline and neurodegenerative disorders where cholinergic signaling is impaired. This mechanism could enhance key cognitive processes such as memory, attention, and learning, further emphasizing its role as a promising natural support for cognitive health.

The neuroprotective effects of Bilberry extract, demonstrated through its ability to protect SH-SY5Y neuronal cells from oxidative stress, are particularly noteworthy (10, 28–30). Oxidative stress, characterized by an imbalance between the production of reactive oxygen species (ROS) and the body’s antioxidant defenses, plays a pivotal role in the pathophysiology of neurodegeneration, mood disorders, and cognitive decline. Prolonged oxidative stress can lead to neuronal damage through lipid peroxidation, DNA damage, and protein oxidation, ultimately compromising neuronal survival and function. The ability of Bilberry extract to mitigate oxidative stress is a critical finding. The robust antioxidant properties of Bilberry extract, as demonstrated in DPPH, ABTS, FRAP, ORAC, and HORAC assays, highlight its capacity to neutralize various forms of ROS and enhance redox homeostasis (10, 12). These effects are attributed to its anthocyanin-rich composition, which enables efficient electron donation and radical scavenging. By alleviating oxidative stress, Bilberry extract can help preserve neuronal integrity and reduce the likelihood of oxidative damage-associated neuroinflammation, a key factor in mood disturbances and cognitive impairment. This antioxidant activity may also support mitochondrial function (31), which is often compromised in oxidative environments, thereby further contributing to neuronal resilience. In addition to its antioxidative actions, Bilberry extract enhances the expression of BDNF, a protein critical for neuronal survival, plasticity, and cognitive function. BDNF is a key mediator in the brain’s response to oxidative stress, helping to repair and regenerate neurons exposed to damaging conditions. The observed upregulation of BDNF in SH-SY5Y cells treated with Bilberry extract under H_2_O_2_-induced oxidative stress underscores its dual role in both reducing oxidative burden and actively promoting neuronal recovery. This dual mechanism provides a strong basis for its neuroprotective effects (10), which may translate into significant therapeutic benefits for conditions characterized by oxidative stress and neurodegeneration.

Importantly, the cytotoxicity evaluation demonstrated that Bilberry extract is safe for neuronal cells, even at high concentrations, supporting its potential for therapeutic applications. This finding aligns with extensive research on Bilberry extract in numerous studies–60 of which have yielded positive results–and in at least 30 controlled or double-blind clinical trials, Bilberry extract has consistently demonstrated excellent safety and tolerability profiles (5–9). Moreover, the safety profile was also supported with the human pilot study. This favorable safety profile, combined with its multifaceted biochemical activities, supports Bilberry extract as a promising supplement for managing low mood and cognitive impairments, conditions often requiring long-term solutions with minimal side effects.

While the findings of this study highlight the promising biochemical and neuroprotective effects of Bilberry extract, it is important to acknowledge the limitations of its *in vitro* design. These results offer valuable insights into the molecular mechanisms underlying Bilberry extract potential benefits for mental well-being, but require validation through clinical studies. Nevertheless, the present study’s emphasis on Bilberry extract neuropharmacological properties strengthens its potential as a reliable, natural support for managing low mood and cognitive impairments, particularly for those seeking safer, long-term adjuvants to conventional treatments. Preliminary evidence in humans obtained from the double-blind, randomized, placebo-controlled Pilot study were consistent with the *in vivo* safety of the 4-weeks Bilberry extract supplementation, and showed beneficial effects on selected mood states evaluated, such as depression, tension and vigor.

It is plausible to hypothesize that the observed effects of Bilberry extract on alleviating low mood and enhancing cognitive performance may be partially attributed to its well-documented ability to improve microcirculation, particularly by enhancing blood flow (10). Previous studies have demonstrated that Bilberry extract can enhance retinal blood flow (7, 10), supporting the concept that improved microcirculation may extend to other tissues, including the brain.

The preliminary findings of this study further support the potential of Bilberry extract to promote mental well-being, providing a possible mechanistic link between enhanced microcirculation and improvements in both mental and physical well-being. Specifically, Bilberry extract was shown to promote mood enhancement, particularly by increasing assertiveness and reducing states of low motivation.

However, given the pilot nature of this clinical study and the relatively small sample size, these findings should be interpreted with caution. Further research with larger, well-powered studies is necessary to confirm the role of enhanced microcirculation in the cognitive and mood-enhancing effects of Bilberry extract and to clarify the underlying mechanisms involved.

## 5 Conclusion

Bilberry extract demonstrates a wide range of beneficial properties, including potent antioxidant, neuromodulatory, vasoprotective, and neuroprotective effects. These findings support its potential as a natural intervention for enhancing mood and cognitive health. The extract’s ability to modulate key biochemical pathways–such as GABAergic signaling, monoamine metabolism, and oxidative stress–highlights its multifaceted mechanisms of action.

This study offers several novel contributions to the current literature. To our knowledge, this is the first report demonstrating the GABA-T inhibitory activity of a standardized bilberry extract. Additionally, we provide an integrated *in vitro* assessment of multiple neuromodulatory mechanisms–including GABA_*A*_ receptor binding, MAO-A, and AChE inhibition–in conjunction with antioxidant assays, linked directly to a pilot human clinical evaluation. This combined mechanistic-clinical approach enhances the translational relevance of the findings and supports the potential of bilberry extract as a multifunctional neuroactive supplement.

The pilot clinical study results, combined with *in vitro* evidence, provide a strong scientific foundation for the potential use of Bilberry extract nutritional supplementation in managing low mood and supporting cognitive enhancement. Specifically, the extract demonstrated improvements in mood parameters, including reduced tension, depression, and confusion, without significant adverse effects. These findings align with its favorable safety profile, further reinforcing its suitability as a natural supplement.

However, given the preliminary nature of the clinical pilot study, further research is warranted. Larger, well-powered clinical trials are needed to confirm these findings, explore the underlying mechanisms in greater detail, and determine optimal dosing strategies. Bilberry extract holds promise as a natural option for promoting mental well-being, and continued research will help establish its role in evidence-based mental health management.

## Data Availability

The original contributions presented in this study are included in this article/supplementary material, further inquiries can be directed to the corresponding authors.
